# Share and protect our health data: an evidence based approach to rare disease patients’ perspectives on data sharing and data protection - quantitative survey and recommendations

**DOI:** 10.1186/s13023-019-1123-4

**Published:** 2019-07-12

**Authors:** Sandra Courbier, Rebecca Dimond, Virginie Bros-Facer

**Affiliations:** 1 EURORDIS-Rare Diseases Europe, Paris, France; 2 School of Social Sciences, Cardiff University, Cardiff, UK

**Keywords:** Rare diseases, quantitative survey, Data sharing, Data protection, Patient engagement, Recommendations, Evidence-based approach, Patient organisation, Public trust, Healthcare, Research

## Abstract

**Background:**

The needs and benefits of sharing health data to advance scientific research and improve clinical benefits have been well documented in recent years, specifically in the field of rare diseases where knowledge and expertise are limited and patient populations are geographically dispersed. Understanding what patients want and need from rare disease research and data sharing is important to ensure their participation and engagement in the process, and to ensure that these wishes and needs are embedded within research design. EURORDIS-Rare Diseases Europe regularly surveys the rare disease community to identify its perspectives and needs on a number of issues in order to represent rare disease patients and be their voice within European and International initiatives and policy developments.

Here, we present key findings from a large quantitative survey conducted with patients with rare diseases and family members as part of a continuous evidence-based advocacy process developed at EURORDIS. The aim of this survey was to explore patient and family perspectives on data sharing and data protection in research and healthcare settings and develop relevant recommendations to support shaping of future data sharing initiatives in rare disease research.

This survey, translated into 23 languages, was carried out via the Rare Barometer Programme and was designed to be accessible to a diverse population with a wide range of education backgrounds. It was widely disseminated via patient organisations worldwide to ensure that a wide range of voices and experiences were represented.

**Main findings:**

Rare disease patients, regardless of the severity of their disease and their socio-demographic profile, are clearly supportive of data sharing to foster research and improve healthcare. However, rare disease patients’ willingness to share their data does come with specific requirements in order to respect their privacy, choices and needs for information regarding the use of their data.

**Conclusions:**

To ensure sustainability and success of international data sharing initiatives in health and research for rare diseases, appropriate legislations need to be implemented and multi-stakeholder efforts need to be pursued to foster cultural and technological changes enabling the systematic integration of patients’ preferences regarding sharing of their own health data.

**Electronic supplementary material:**

The online version of this article (10.1186/s13023-019-1123-4) contains supplementary material, which is available to authorized users.

## Introduction

We live in an information age with exponential increases in biomedical information available to support scientific advances and inform health care decisions. These developments are propelled by ‘omic’ research - genomics, transcriptomics, metabolomics, proteomics, etc. – made possible through the increased technical capacity to produce and store large datasets, amidst decreasing technical costs [39, 44]. This move towards ‘big data’ has significant implications: the capacity to analyse collective biomedical information raises significant and challenging questions on how to exploit its potential while protecting the interests of individual contributors and stakeholders. Within this current landscape, there is an imperative to make effective and timely use of the data to ensure translation towards improvement in clinical outcomes. It is now broadly recognised that this is only possible through collective endeavours: the true potential of biomedical data can only be realised through its capacity to be combined and shared. Sharing data - rather than data operated in isolation from others - is now recognised as one of the most important ways to ensure benefits for all, including patients, families, scientists, funders, health care providers and future users of the healthcare systems. The basic principal behind data sharing is that the scientific community should, wherever possible, pool their data to gain the maximum benefit from it; this would be, for example, combining two or more datasets from researchers working in the same area, to make one large dataset, which then becomes more statistically significant.

High profile initiatives, focusing on both ‘healthy’ and disease-linked populations attest to the value of embedding data sharing in their design, and this is particularly evident for projects exploiting the potential of new genetic technologies which are propelling the big data revolution. The Human Genome Project, completed in 2003, which succeeded in mapping the human genome, was only possible through widespread international collaboration. A more recent example is the UK 100,000 Genomes Project, which was launched by Genomics England with the aim to sequence 100,000 genomes of NHS patients with the intention to support scientific and clinical advances, and to create an NHS genomic medical service.

Shared informational data enables a much deeper and broader understanding of the nature of disease and patient populations than was previously possible. It can provide a greater evidence base for improving clinical outcomes, informing clinical trials, and supporting the development of drugs and devices. It can also improve and speed up the diagnostic process, improve its accuracy and consequently reduce health costs. Overall, there are clear financial benefits in terms of reducing duplication and speeding up the process from bench to bedside. For example, Figueiredo [20] argues that data sharing is a way of returning the investment that society has made in science through public funded research or charity. As a consequence, sharing data is imperative in the context of rare disease research. As most rare diseases have a genetic component, clinical benefits are now possible through the developments of genomic technologies, yet sharing rare disease data is further complicated by the nature of rare disease. There are more than 6000 rare diseases which represents great biomedical and clinical variability. Low prevalence rates, few if any centres of expertise and wide geographical spread can make it difficult to identify adequate numbers of patients at a local level to inform a valid evidence base. Thompson et al. [44] highlight an example of a trial for juvenile dermatomyositis, where 103 clinical centres were involved in recruiting 130 patients. Data sharing within, and across rare disease groups can produce knowledge more efficiently by minimising the potential for duplicated studies, but also reducing the burden of research on small yet willing patient communities [11].

The role of patient communities has been well documented in raising awareness of little known medical conditions and campaigning for access to research funding and healthcare resources [2]. Patients with a rare disease are increasingly willing to engage with research as it often offers the only hope of accessing a diagnosis or benefitting from a treatment or a cure. Yet the willingness of patients and their families to support the scientific research agenda and engage with biomedical research and data sharing can leave them vulnerable. One of the problems with rare diseases has been that the hope and promises associated with developments in technologies have often been slow to translate into clinical outcomes, and that while there might be scientific merit, patient communities have often not experienced any benefit. At the extreme end, some have felt exploited in the race for scientific gain (see for example the problems within the research partnership focusing on Canavan Disease, as documented in Novas 2006).

This article contributes to the debate by identifying key issues about data sharing, enabling support for research while ensuring that participants are protected. Understanding what patients want from rare disease research and data sharing is important to ensure their participation and engagement in the process, and also to ensure that consideration of their needs are embedded within research design.

Although the benefits of sharing data are clear, there are numerous technical and regulatory boundaries which make sharing difficult and for many researchers, clinicians and institutions, still not standard practice. Data sharing requires a level of compatibility which can be difficult to implement in practice. With preferred systems and protocols, practices which dictate what kinds of data can be collected and what can be done with it, these multiple systems, owned or managed by different institutions, different countries and different regulatory rules can be incompatible. However, aside from technical issues, there is a different kind of barrier noted by Contreras and Reichman [8] and others, that many institutions do not have a ‘culture’ of sharing data which would make introducing new data sharing initiatives more difficult. Many researchers work closely within geographic, institutional or disciplinary boundaries. In the context of ‘silo mentalities’, sharing data is replete with concern about risking the personal and professional benefits gained through ‘ownership’ of data. The American College of Medical Genetics and Genomics position statement [1] identified a ‘pre-competitive space’ where the benefits of sharing could be widely distributed. Although the College recognises that sharing of data is vital for ensuring genetic health care and securing improved clinical outcomes, it suggests that this will require a ‘paradigm shift’ in research and practice. Conflicting needs of contributors have been addressed by introducing an embargo period which protects the interests of scientific lead partners while ensuring that data can remain open and available to others [7].

The challenges faced by investigators in relation to data sharing are compounded by the hurdles inherent in rare disease research, including investigators’ lack of knowledge and understanding of the context of rare disease, difficulties in accessing funding and developing new treatments [25]. Although Griggs et al. [25] are right to some extent in noting that rare disease patients are often willing research participants, there are many barriers to participation which need consideration. Key issues associated with data sharing, such as consent, anonymity and trust are important not only because they offer participants a level of protection in the research, but also because the way these issues are managed has significant implications for patients’ perspectives on research and their willingness to engage.

Thompson et al. [44] highlight how the risks of collecting, storing and manipulating large data sets are informational rather than physical. With much publicised cases of data hacking, data security is of course paramount, but there are more fundamental issues to consider: the production and availability of digital biomedical data has created concerns around privacy and consent, as well as ownership and control.

Understanding issues of consent within any biomedical field is often a priority for researchers. Yet informed consent can be rendered meaningless because of the complexity of the research and its purposes. This is particularly so in relation to the genomic revolution, where there is a long history of acknowledging complex issues around consent, and where there is often an acknowledged lag between research and treatment - the multifarious purposes for collecting and storing biomedical information compare with medical research with more tangible and local clinical applications. Consent around genetic medicine for example is complicated by the importance of collecting family data [42] and gaining consent from children [6, 31]. It also has the potential for incidental findings [27] and where future developments in technology and diagnostic capacity might mean re-contacting patients with new information [40].

Different models of consent which have been identified specifically in relation to rare disease patient communities include broad, dynamic and opt-in. Gainotti et al. [22] for example explored issues around patient consent for those involved in international collaborative rare disease research, and highlight, as others have done, the pressing need for advanced planning. They make a distinction between data that has already been collected (and which is bound to already received consent and its limitations) and new research which can be collected under new guidelines. They conclude that for newly collected data, “broadly described research purposes with ongoing updates for participants is the best current solution” ([22] p1253). They see this as allowing the flexibility to adapt to new circumstances and technologies but also one that protects participants and ensures transparency.

McCormack et al. [31] found that patients valued being asked to re-consent when a research purpose changed. They stated that consent is “a social agreement”, and decisions about research are not automatically conferred to the research team or ethics panel. Current practice within many countries and institutions has often been based on the premise that data re-use is less ethically contentious when it can be anonymised [11]. Anonymity is a complex issue in the case of biomedical information, made more difficult for rare disease patients because the risks of identification are higher due to the much smaller populations and made even more complex because of the frequent requirement to collect family data. The information which is important for characterising the biological nature of a particular rare disease are precisely the kinds of information which can identify the patient. With such small populations, identifying the name of the disease and the country of residence might be sufficient to identify an individual patient. Although discrimination on the basis of genetic information is regulated in the US (with the adoption of the Genetic Information Nondiscrimination Act of 2008) as well as across many EU countries, other countries have yet to initiate discussions about the risks associated with genetic knowledge. Offering rare disease patients the opportunity to have feedback, while protecting their identity through anonymity remains a challenge.

Rare disease patients’ perspectives are needed to contribute to the debate on the management, sharing and protection of data, in order to reconcile tensions within the research process with what matters most to patients. McCormack et al. [31] found that patients are aware of and concerned about questions of data security and misuse. They also recognised their vulnerability associated with having a rare disease and that knowledge generated through genomic developments and data sharing could lead to their discrimination.

The dominant picture which emerges from current research is that patients are willing to contribute their data but continue to have concerns about data sharing. The European Commission survey programme (Special Eurobarometer, 2018, [14]) for example identified that a sense of control is important for citizens involved in research, yet currently they often do not feel in control of what happens to their data. Trust is a key issue for patients involved in rare disease research, and it could be argued that this becomes even more evident in data sharing, with the onus on researchers, institutions and collaborations to recognise this as a responsibility. Focusing on those with a rare disease, McCormack et al. [31] report that “participants have high levels of trust in public institutions” and they expect that institutions will, and should, recognise their right for their privacy, and their data to be protected and used effectively. Darquay et al. [10] found similar results, that patients (in their case members of a European leukodystrophies database) supported data sharing in order to generate greater knowledge and clinical outcomes. Patients wished for continued information and transparency, they demonstrated trust in the researchers and ethics committees to protect their interests, but most importantly, to enable research to progress.

Supporting continuous and open communication with participants might be onerous for researchers and collaborators, but it recognises the crucial role that participants play within the research process. Including patients at the design stage of projects can ensure the feasibility of research protocols, and can help ensure its success [33]. More importantly, understanding the perspectives of patients and engaging them in the process is key for promoting and maintaining trust within the patient-research relationship, and highlights the importance of solidarity, reciprocity and co-production [34]. There are also issues about recruitment and retention. Researchers continue to stress the importance of informed consent, even though opting out could be problematic for research given such low numbers and the high value of participation. Gainotti et al. [22] for example underlines the crucial importance of supporting patients in expressing their informed consent and allowing the time, care and resources that this requires. A major concern linked to rare disease research, given the significance of participation is that, as the technology develops, or new purposes or collaborations are found, re-gaining consent risks losing participants at each stage. Parallels can be drawn with the experience of recruitment and retention of minority research participants, where distrust is a significant barrier, and where community involvement and ongoing communication can support engagement.

The present survey and suggested recommendations are specifically relevant today in view of wider changes in regulation and attitudes towards data. The implementation of the General Data Protection Regulation (GDPR) engenders a greater awareness of the value of data, issues of ownership and privacy and highlights potential risks to participants [43]. Haeusermann et al. [26] explored the reasons why individuals shared their own personal health data online. They found that participants who were openly sharing their own data continued to have concerns about privacy, and that the motivation for sharing despite this risk was that it could lead to new developments. But they identified that choosing to make public their private data was based on a knowledge of, and compromise around, the risks and benefits. Thus a contradiction has emerged, between the widespread use of social media and a greater freedom in sharing data, amidst rising concerns about privacy [38].

The International Rare Diseases Research Consortium (IRDiRC) was established in 2011 in an effort to support global collaboration on research for rare diseases. RD-Connect was one of the first projects to be funded under the IRDiRC initiative (see [44]). RD-Connect is a large EU-funded project aiming at developing an integrated platform connecting databases, registries, biobanks and clinical bioinformatics for rare disease research [31, 44]. EURORDIS-Rare disease Europe is actively involved in IRDiRC activities as well as in RD-Connect, including the coordination of a Patient Advisory Council (PAC) to inform all project partners of issues important to patients and guaranteeing a patient-centric approach throughout the various activities [29]. This survey forms part of a broader mixed methods approach to exploring perspective on data sharing and protection that was initiated through RD-Connect. The findings of related qualitative projects conducted through RD-Connect have previously been published [31]. Indeed the present quantitative survey represents an important contribution in providing a more detailed picture of the experiences and opinions of those living with a rare disease.

The present study is part of a continuous evidence-based advocacy process developed at EURORDIS. Evidence-based advocacy is generally described as involving the use of research to influence policy [9, 28]. Evidence refers to a result or output of a research process that can include any process of investigation such as data collection, analysis and codification that can be used to inform policies. Evidence-based initiatives seek social transformation by advocating for the rights of the most vulnerable [28]. As a European umbrella of rare disease patient organisations with over 800 members, part of EURORDIS’ mission is to represent rare disease patients and be their voice within European initiatives, projects and policy developments that affect their daily lives. Requests for patient perspectives in health, research and social policy-making are on the rise as the benefits associated with evidence-based programmes or policies, as described by Brownson [4], are being increasingly recognised and required by all stakeholders. The Rare Barometer programme is an initiative that uses social science research methods to collate and examine the perspectives of patients living with a rare disease and their family, ensuring their accurate representation in EURORDIS’ advocacy work. The Rare Barometer programme hosts an international survey panel known as Rare Barometer Voices, which was designed to address the difficulties of making and sustaining contact with people living with a rare disease. Members of the Rare Barometer Voices panel agree to regularly take part in surveys. It is also designed to ensure diverse representation. Diversity is achieved by recruiting through various sources, including rare disease organisations, social care providers, Google and Facebook adverts. Information is provided through online channels (i.e. Facebook, twitter, emails) and other means of communications (e.g. leaflets distributed during rare diseases-related events). When individuals register for Rare Barometer Voices, they will be asked to provide socio-demographic information such as age, gender and country of living. The Rare Barometer Programme aims to collect rare disease patients’ perspective on a variety of topics to provide general guidance and strategic information on relevant policies for rare disease patients and their families. This programme works towards identifying priorities and problems [4] within the rare disease field, and measure their magnitude and seriousness. It contributes to policy-agenda setting at EURORDIS and to suggest alternative or more targeted policy approaches that may be taken to address difficulties encountered by rare diseases patients. In 2017 for example, within the framework of the European Union-funded INNOVCare project (https://innovcare.eu), results of a survey among 3071 rare disease patients have served to assess unmet social needs of people living with a rare disease [17] which then contributed to the design of an innovative care pathway bringing together national resource centres for rare diseases and case managers.

## Materials and methods

This article presents findings from a large quantitative survey conducted with patients with rare diseases and family members from March to May 2018. The aim of this survey was to explore patients and family perspectives on data sharing and data protection in research and healthcare settings. It was designed to be accessible to a diverse population with a wide range of education backgrounds: the survey was translated into 23 languages to make it accessible to non-English speakers, and was disseminated via patient organisations to patients who are not necessarily used to taking a position on a data-related policy issues, thus ensuring that a wide range of voices and experiences were represented.

There were three main objectives of this quantitative study. The first was to gain a better understanding of the opinions, expectations and concerns about data sharing of patients with a rare disease and their family members. Secondly, it was hoped that the findings of this project would complement previous work on patients’ perspectives conducted through RD connect, either through confirming or refuting their main findings. Finally, it was expected that this project would lead to policy and research recommendations, to encourage researchers and healthcare stakeholders in charge of or participating in data-sharing initiatives to recognise the importance of understanding rare disease patient perspectives, and to encourage discussion about data sharing best practices.

The target population was patients living with a rare disease or family members (parents and close relatives) of over 16 years old. Fieldwork started in February 2018 and ended in April 2018. Rare Barometer Voices participants received an email to inform them about the launch of the survey and inviting them to take part. Those who did not reply received 4 reminders. One thousand three hundred sixty-four participants completed the questionnaire, yielding a response rate of 19%, which is comparable to similar studies.

Information about Rare Barometer Voices containing a link to the survey was also disseminated on social media and through the EURORDIS network of patient organisations, and 871 responses were received. Two thousand two hundred thirty-five responses were collected in total. Questionnaires completed to more than 80% only were kept, duplicates and responses from respondents outside the target population were excluded from the analysis. Two thousand thirteen responses were analysed. For more information on the repartition of the population see Additional file 1 about the profile of the respondents, Additional file 2 about the relationship with rare diseases, Additional file 3 about the repartition by group of diseases, Additional file 4 about diseases with more than 20 respondents included in the sample, Additional file 5 about the repartition by country.

The questionnaire was designed in consultation with a topic expert committee representing diverse expertise: sociology, legal, computational biology, rare disease patient advocacy, ethics, patient reported outcome measurement and rare disease advocacy. It was also influenced by insights gained through the qualitative exercises linked to RD Connect [44]: focus groups exercise [31], Delphi exercise with 15 knowledgeable patients aiming at reaching consensus on data-sharing-related issues, group discussions with members of the Patient Advisory Council (PAC) of the RD Connect project. Particular questions were also influenced by previous quantitative surveys which have specifically focused on data sharing and data protection, including for example the European Commission survey programme (Special Eurobarometer, [13, 14]), which allows comparison between rare disease patients’ perspectives and those of the general public.

The questionnaire was mainly composed of close-ended questions with defined response categories, addressing the following areas:

- The degree of sensitivity attributed to different types of health-related information;
- The trust placed in different stakeholders that could be involved in data sharing initiatives;
- The different purposes for which they would be willing to share their data;
- The type of information rare disease patients would need to receive to engage in a data sharing project;
- The ways and the frequency to which they would want to receive information of the related projects;
- The degree of control they would want to have over their data; and.
- The risks associated with the potential disclosure of health data.

Descriptive statistics of responses are expressed in percentages. In order to improve readability and comprehension, most of the response categories and Likert scale items are grouped. In order to investigate sociodemographic factors associated with responses related to these areas, questions regarding sociodemographic profile and behaviour were included in the questionnaire and used as independent variables. These included gender, age, age at end of education, relationship to rare diseases (patient, carer, patient representative), use of social network, severity of the disease and perceived sensitivity of data. The Chi-Square test of independence was used to assess if there are significant differences between subgroups [32]. When relations between dependent and independent variables were not significant (based on chi squared test) and *p* value above 0.05, results were not included in the description of the results. MAPI, partner of the Rare Barometer Programme and expert in medical translation and Linguistic Validation, provided the translation.

## Results

### Respondents widely support data sharing if done in the interest of rare disease patients

Almost all respondents would be willing to make their own health data or that of the person they care for available for research purposes, whether it is used to develop new treatments (97%), to improve research on diagnosis (97%) and/or to better understand mechanisms and causes of the disease (97%). A vast majority of the respondents are also willing to share their data in healthcare settings, 95% being in favour of sharing their data to receive additional specialist advice on their care. An overwhelming majority would also be keen to share their data to improve research on other diseases (90%) (Table 1).

**Table 1:** If given the opportunity, would you be willing to make your/the person you care for health information available for the purpose of

(*n* = 2013)	Number of people	% of responses
Better understanding mechanisms and causes of your disease		
Yes^a^	1954	97%
No^b^	40	2%
Don’t know	19	1%
Developing new treatments for your disease		
Yes^a^	1953	97%
No^b^	41	2%
Don’t know	19	1%
Improving diagnosis of your disease or suspected disease		
Yes^a^	1946	97%
No^b^	44	2%
Don’t know	23	1%
Receiving additional specialist advice on your care		
Yes^a^	1915	95%
No^b^	76	4%
Don’t know	22	1%
Improving research and care on diseases other than yours		
Yes^a^	1803	90%
No^b^	166	8%
Don’t know	44	2%
Carrying out research not related to the medical field		
Yes^a^	1029	51%
No^b^	841	42%
Don’t know	143	7%

^a^Includes those who responded either ‘yes, definitely’ or ‘yes, probably’

^b^Includes those who responded either ‘no, probably not’ or ‘no, definitely not’

Because of rounding, percentage might not add up to exactly 100%

The willingness to share data for the above purposes is shared across all socio-demographic categories (age, gender, level of education, severity of the disease), which shows a high level of consensus on the idea of sharing data for care-related matters. Only respondents aged 65 and over are slightly less likely to share their data to improve research on diseases other than theirs (84%).

However, only a small majority of the respondents (51%) are in favour of sharing their data for purposes not related to the medical field. The socio-demographic data show that respondents over 50 years old are less open to the idea of sharing data outside the medical field (45%) whereas the majority of respondents under 50 years old (55%) would be willing to share their data for this type of purpose. In addition, respondents with lower level of education are more open to sharing for non-medical purposes (59%) than those with higher levels of education (48%). Rare disease patients with more severe diseases are more disposed to share their data for non-medical purposes (64%) compared to those with less severe diseases (40%). Looking at country variations, it also appears that respondents from countries belonging to the European Union are less favourable (50% compared to 60% outside the EU) to share data for non-medical purposes. Lastly, respondents who are not active users on social media (who do not share information online everyday) are also less keen to share data outside the medical field: 43% compared to 54% among active users (who share information online everyday).

### Why participate in rare disease research? The possibility to discuss and learn about the rare disease are the main incentives for patients

Respondents were asked to choose elements that would encourage them to participate in data sharing projects among a list of seven elements. From this list, all items related to having the possibility to receive more information and to learn more about their rare disease were the most quoted, about three times more than other items on the list. 69% think that the possibility to learn more information about the disease would encourage them to participate, 66% chose the possibility to discuss and ask questions directly to professionals as their main incentive and 62% opted for the opportunity to be informed on the results of the project (Table 2).

**Table 2:** From the list below, what are the three main options that would encourage you to participate in a project involving the sharing of your/the person you care for health information? (Please select the responses in priority order)

(*n* = 2013)	Number of people	% of responses
The possibility to learn more information about your disease	1382	69%
The possibility to discuss and ask questions directly to professionals involved in the project	1322	66%
The possibility to be informed on the results of the project	1251	62%
The possibility to access your health information	541	27%
The option to withdraw the information at any point during the project	505	25%
Being sure to be contacted if your information has been misused	478	24%
Having the time to process the information and decide at a later stage on whether you want to participate	343	17%
Other	46	2%
I wouldn’t give the possibility of sharing these health information	28	1%
Don’t know	25	1%

Several answers possible, so percentage does not total 100%

Sociodemographic data show that respondents residing in countries outside the European Union are particularly interested in learning more information about their disease: 73% think that the possibility to learn more information about the disease would encourage them to participate compared to 68% for those living in the European Union. The need for information varies across diseases from 45% of carers or patients affected by cystic fibrosis to 81% of patients or carers affected by systemic sclerosis (although the results should be considered with caution because of the variable and sometimes low number of respondents per disease).

Having the possibility to discuss the disease is of particular importance for carers (69% compared to 64% for patients) and this importance also varies across diseases, from 44% for sarcoidosis to 76% for myasthenia gravis.

Following the importance of gaining more information on the rare disease; options that would give patients and carers the possibility to have more control over the data are selected by about one quarter of the respondents: 27% declare that having the possibility to access their health information would encourage them to participate, 25% chose the possibility to withdraw their data at any time of the project- this option being more important for patients with higher level of education (27%) than those with lower level of education (18%) - and 24% opted for the option to be contacted if their information has been misused. The need to be recontacted in that case is a more important element for respondents living in the European Union (25%) than for respondents living outside the European Union (17%). Lastly, the possibility to decide at a later stage if they want to participate is quoted by only 17%.

### Opinions are divided on the sensitivity of different types of health information

Respondents were asked to report the level of sensitivity they associate to several types of health data. Respondents show mixed views about these levels of sensitivity: roughly half of them think that information on their disability (51/47), genetic information on their disease (49/48), physiological data (48/50) are sensitive (very or fairly sensitive). Symptoms (42/57) and names of the disease (33/65) are perceived as not sensitive (not very sensitive or not sensitive) by a majority of the respondents (Table 3).

**Table 3:** Imagine you are asked to participate in a project that involves sharing your/the person you care for health information. In this context, how sensitive do you think each of the following types of information are?

(*n* = 2013)	Number of people	% of responses
Information on your disability		
Sensitive^a^	1022	51%
Not sensitive^b^	944	47%
Don’t know	47	2%
Genetic information on your disease		
Sensitive^a^	986	49%
Not sensitive^b^	961	48%
Don’t know	66	3%
Physiological data (e.g. blood pressure, results of biological analysis)		
Sensitive^a^	960	48%
Not sensitive^b^	1005	50%
Don’t know	48	2%
Symptoms of your disease		
Sensitive^a^	838	42%
Not sensitive^b^	1141	57%
Don’t know	34	2%
Name of your disease		
Sensitive^a^	664	33%
Not sensitive^b^	1315	65%
Don’t know	34	2%

Because of rounding, percentage might not add up to exactly 100%

^a^Includes those who responded either ‘very sensitive’ or ‘fairly sensitive’

^b^Includes those who responded either ‘very not sensitive’ or ‘not sensitive’

The socio demographic analysis shows that older respondents (over 50 years old) tend to see all categories of health-related information -except information on disability- as more sensitive (53% for genetic information, 50% for physiological data, 44% for symptom of the disease, 35% for the name of the disease) than younger respondents (below 50 years old: 47% for genetic information, 46% for physiological data, 40% for symptom of the disease, 32% for the name of the disease). Women (52%) perceive information on disability as more sensitive than men (46%). The perceived sensitivity of any types of the above information - except information on disability - is higher among respondents who present themselves as patient representatives (59% for genetic information, 54% for physiological data, 50% for symptom of the disease, 42% for the name of the disease).

Genetic information is of particular concern among patient representatives: 35% think that genetic information is *very sensitive* (as it appeared in the questionnaire) compared to only 27% of carers and 23% of patients.

Looking at the results of this question as a dependant or explanatory variable also shows that the willingness to share data is very lightly affected by the perceived sensitivity of data: respondents who perceive all type of the above health-information as sensitive are more than 90% to be willing to share their data for each health-related purposes.

Respondents who think their data is sensitive require more control over their health information (54%). Results also demonstrate that the willingness to share and to control data are not contradictory: respondents who are used to share information online are also asking for control over their data (45% of those who use social network provide a grade of 5 – full control). This is scored even greater among those who never share information online (56%).

### Patients want to keep control over the data they are sharing

Being in favour of sharing their data does not preclude respondents from wanting to keep control on the whole data sharing process. On a scale from 1 to 5 on which 1 represents having no control and 5 having the full control over their data, almost no respondent declare that they do not request any control over their data (1%). Respondents are overwhelmingly in favour of keeping the strictest control on their data: 47% choose a grade of 5 and 33% a grade of 4 (Table 4).

**Table 4:** Still in the situation in which you/the person you care for are sharing your health information. On a scale from 1 to 5, how much control would you like to have over this information?

(*n* = 2005)	Number of people	% of responses
1 - No control (on who, how and why using your information)	21	1%
2	77	4%
3	301	15%
4	671	33%
5 - Full control (on who, how and why using your information)	935	47%

Because of rounding, percentage might not add up to exactly 100%

Looking at sociodemographic data, women (48% selected give a grade of 5) are more prone to request control over their data than men (42%). Respondents residing in the European Union are also in favour of more control (48% selected grade 5) than respondents coming from countries outside the European Union (37%).

### The uses of data under unchosen circumstances are the mains risks associated with sharing data

In line with the importance attached to controlling their data, rare disease patients are concerned that their data could be used by third parties with which they would not have chosen to share their data (50%). For most this is the most prominent risk they associate with the disclosure of their personal data. They are almost equally concerned that their data could be used in a context they would not have chosen (47%). The third risk would be to see their information being used without being aware of it (35%).

More than one third of the respondents are apprehensive of becoming victims of discrimination (34%) as well as their identity being used for fraudulent purposes (32%). Uses that could produce direct and harmful consequences such as becoming victim of a fraud (20%) or their personal safety being at risk (12%) are less seen as potential risks for rare disease patients than the previous ones (Table 5).

**Table 5:** Below is a list of potential risks. According to you, what are the most important risks connected with disclosure of your personal or health information? (Please select three responses in priority order)

(n = 2013)	Number of people	% of responses
Your information being shared with third parties (companies or government agencies) without your consent	978	50%
Your information being used in different context from the ones where you disclosed it	915	47%
Your information being used without your knowledge	683	35%
Becoming the victim of discrimination (e.g. in job recruitment, being charged higher prices, not being able to access a service)	662	34%
Your online identity being used for fraudulent purposes	620	32%
Your information being used to send you unwanted commercial offers	407	21%
Becoming a victim of fraud	392	20%
Your personal information being stolen	348	18%
Your personal safety being at risk	314	16%
Your views and behaviours being misunderstood	177	9%
Your reputation being damaged	100	5%
Your personal information being lost	89	5%
I wouldn’t give the possibility to share my health data	28	1%
None	19	1%
Other	11	1%

Several answers possible, so percentage does not total 100%

### Rare disease patients show higher level of confidence in not-for-profit stakeholders

Respondents were asked whether they trust various authorities and type of organisations to handle and use their health information carefully. Trust in not-for-profit stakeholders (89% for medical doctors, 79% researchers from non-profit organisations, 77% for patient organisations, 69% for healthcare professionals other than medical doctors) is considerably higher than trust in for profit stakeholders. Among stakeholders from the not-for-profit sector, medical doctors involved in the respondents’ healthcare are the most trusted (almost 9 in 10 respondents). Similarly, confidence in patient organisations is very high (77%). Patients representatives show a high level of confidence (83%) toward this type of organisation, but so do patients (83%) and carers who do not identify themselves as representative (76%). Opinions on governments and institutions from the respondents’ country are more divided (48% show confidence compared to 47% who show little confidence), but more confidence is stated towards governments and institutions from the European Union (51%) than from other countries (31% compared to 61%). Cross-analysis shows that patient representatives tend to trust their government (60% confident compared to 38%) more than patients (47% confident compared to 49% not confident) or carers (54% confident compared to 43% not confident) who are not engaged in advocacy activities. Sociodemographic tables also show that more educated respondents (those who finished education when they were 20 or more) tend to trust government and institution from their country more (53%) than those who finished school earlier (44% among those who finished school before 20 years old) (Table 6).

**Table 6:** Imagine you are still in a situation in which you are asked to participate in a project that involves sharing your/the person you care for health information. People involved in the project can belong to different types of organisations. How much confidence do you have in each of them to handle and use your health information carefully?

Medical doctor involved in your healthcare	Confidence^a^	1795	89%
	Little confidence^b^	198	10%
	Don’t know	20	1%
Researcher from a non-profit organisation (e.g. university or public hospital)	Confidence^a^	1592	79%
	Little confidence^b^	360	18%
	Don’t know	61	3%
Patient organisation	Confidence^a^	1546	77%
	Little confidence^b^	391	19%
	Don’t know	76	4%
Healthcare professionals other than medical doctors (e.g. dentists, pharmacists, nurses, physiotherapists)	Confidence^a^	1381	69%
	Little confidence^b^	571	28%
	Don’t know	61	3%
Government or institution from a country within the European Union	Confidence^a^	1017	51%
	Little confidence^b^	860	43%
	Don’t know	136	7%
Government or institution from your country	Confidence^a^	988	48%
	Little confidence^b^	946	47%
	Don’t know	79	4%
Researcher from a private genetic testing company	Confidence^a^	956	47%
	Little confidence^b^	936	46%
	Don’t know	121	6%
Researcher from a pharmaceutical or medical device company	Confidence^a^	911	45%
	Little confidence^b^	1010	50%
	Don’t know	92	5%
Government or institution from a country outside Europe	Confidence^a^	625	31%
	Little confidence^b^	1219	61%
	Don’t know	169	8%
An insurance company	Confidence^a^	315	16%
	Little confidence^b^	1619	80%
	Don’t know	79	4%

Because of rounding, percentage might not add up to exactly 100%

^a^Includes those who responded either ‘a great deal’ or ‘quite a lot’

^b^Includes those who responded either ‘not very much’ or ‘none at all’

Regarding the private sector, opinions are divided about researchers working for the pharmaceutical industry (45% are in favour and 50% are opposed). However, a large majority of the respondents are opposed to sharing their data with insurance companies (16% are in favour and 80% are opposed). Sociodemographic analysis shows that the older respondents are less likely to trust the private sector: 57% of respondents under 25 trust researchers from pharmaceutical industry compared to 36% for respondents over 65 and only 28% compared to 9% for insurance companies.

### Opinions are fragmented on whether responsibility could be delegated to an ethics committee

A relative majority (49%) would allow an ethics committee to decide on their behalf with whom their information could be shared, 43% are against the idea and 8% do not have an opinion. The sociodemographic data shows that men (58%) are more disposed to let an ethics committee decide for them than women (46%). Willingness to delegate responsibility to an ethics committee corresponds with increasing age. Respondents over the age 50 were more ready to delegate decisions (52% among respondents aged 50 to 64 and 59% among 65 and older) compared to younger respondents who are willing to delegate (40% under 24, 42% between 25 and 34 and 48% between 35 and 49). People residing outside the European Union are more willing to delegate to an ethics committee (67%) than those living in the European Union (46%) (Table 7).

**Table 7:** Would you allow an ethics committee to decide on your behalf with whom your information will be shared, how and why?

(*n* = 2005)	Number of people	% of responses
Yes^a^	980	49%
No^b^	863	43%
Don’t know	162	8%

Because of rounding, percentage might not add up to exactly 100%

^a^Includes those who responded either ‘yes, definitely’ or ‘yes, probably’

^b^Includes those who responded either ‘no, probably not’ or ‘no, definitely not’

### Communicating with patients

When asked directly if they would like to be informed about the outcome of a data-sharing project in which they are participating, almost 100% of the respondents (99.7%) answer positively. This percentage is higher than the one presented earlier in the results chapter because is it not presented to respondents in competition with other items (62% opted for the opportunity to be informed on the results of the project, see Table 2).

### Knowing about the outcome and understanding the main features of the project are the most important information for patients to receive

Receiving details on how the project could be beneficial to their disease or other diseases is the most important piece of information for the respondents (80% of the sample selecting this option, compared to much lower percentages selecting the rest of the possibilities). Around half of the respondents want to receive an easily understandable summary of project (51%) and information about the management rules (49%). Around 40% want to know about the professionals involved in the project and how these professionals might benefit from the project. Information on measures taken to prevent security breaches are selected by less than one third of the respondents (28%) (Table 8).

**Table 8:** From the list below, what are the three main pieces of information about the project that would be important for you to receive? (Please select three responses in priority order)

(*n* = 2013)	Number of people	% of responses
Detail on how the project could be beneficial for your disease or other diseases	1605	80%
Brief summary of the key information necessary to understand the main aspects of the project	1032	51%
Information about the data management rules (ie. how access to my health information will be granted or is there an ethical review?)	988	49%
Information about professionals involved in the project who can access the health information (e.g. their biography)	797	40%
Information on how professionals involved in the project might benefit professionally from accessing my health information	750	37%
Information on the measures taken to prevent security breaches	561	28%
Don’t know	64	3%
I wouldn’t give the possibility of sharing these health information	28	1%
Other	26	1%

Several answers possible, so percentage does not total 100%

The hierarchy of importance does not vary significantly across socio-demographic categories.

### Respondents favour most common way of receiving information such as emails or face to face discussion

About 9 in 10 of the respondents would like to be informed by emails or during face-to-face discussions, these opinions are equally shared across socio-demographic categories. 85% would like to receive information through a dedicated website, but this option is favoured by respondents who are active users on social media (89%) compared to those who are not social media users (77%). Almost 7 in 10 would be prepared to attend a conference to learn about the project in which their data is involved, which is less than other items but still represents a large majority of the respondents. Opinions on receiving information through a mobile app are more divided. Respondents below the age of 50 (59% under 24, 70% between 25 and 34) are much more open to consulting a mobile app than respondents over 50 (49% among 50–64, 37% among 65 years old and older). Respondents coming from countries outside the EU are also more disposed to receiving information through a mobile app (62%) compared to EU residents (55%) (Table 9).

**Table 9:** Would you like to be informed about the outcome of the project through each of the following means?

(*n* = 2005)	Number of people	% of responses
Emails		
Yes^a^	1863	93%
No^b^	127	6%
Don’t know	15	1%
Face to face discussion with professionals involved in the project		
Yes^a^	1718	86%
No^b^	235	12%
Don’t know	52	3%
A dedicated website		
Yes^a^	1706	85%
No^b^	250	12%
Don’t know	49	2%
Attending conferences		
Yes^a^	1349	67%
No^b^	558	28%
Don’t know	98	5%
A mobile app		
Yes^a^	1119	56%
No^b^	819	41%
Don’t know	67	3%

Because of rounding, percentage might not add up to exactly 100%

^a^Includes those who responded either ‘yes, definitely’ or ‘yes, probably’

^b^Includes those who responded either ‘no, probably not’ or ‘no, definitely not’

The ideal frequency to be informed for a majority of the respondents is once a month (55%). It can also be noted that some respondents would favour more frequents updates (21%), in particular respondents under 35 years old (31% among under 24 and 27% among 25–34 years old) and respondents coming from outside the European Union (31%) (Table 10).

**Table 10:** And how often would you like to be informed about the outcome of the project?

(*n* = 2005)	Number of people	% of responses
Several time a week	112	6%
Once a week	427	21%
Once a month	1101	55%
Once a year	241	12%
Don’t know	124	6%

## Discussion

The results of this large quantitative survey strongly substantiate previous findings among a wider rare disease patient population. Rare disease patients and representatives, regardless of the severity of their disease and their socio-demographic profile, are clearly willing to share their data to foster research and improve healthcare. Results also show that the perceived sensitivity of data does not prevent rare disease patients to be willing to share them. This aligns with qualitative work focusing on the perspectives of rare disease patients on data sharing which showed that patients are positively disposed towards participating in research and allowing their own data or data from family members they care for to be shared internationally. Compared to the general population, rare disease patients seem to be more inclined to share their data. In a study carried out by YouGov in 2018 [3] in several European countries and among the general population, only 37% of the respondents declare that they would be ready to share their data to develop medicine and treatments. The support for data sharing in the context of rare disease research is aligned with the work undertaken by the European Reference Networks to establish a dedicated data-sharing platform enabling information exchange and mutual learning to improve rare disease patients’ diagnosis and care, while also contributing to the development of research and innovation. A politically significant initiative was launched last year, with the signature by several EU countries of a declaration whereby their governments commit to cooperate to deliver cross-border access to genomic information. This declaration has the potential to maximise use of health care resources and advance the development of personalised medicine especially in the rare disease field [12].

Recently, the European Commission released a recommendation to securely share electronic health records across Europe, building on existing programs to share e-prescriptions and patient summaries. The Commission wants to create a framework for an EU-wide exchange platform where national systems would be able to exchange information. The potential impact of this recommendation will be entirely dependent on the willingness of countries to make the necessary investments in their national health IT infrastructure [16].

Electronic health records do not yet exist in most EU countries for a number of reasons including lack of interoperability, fragmentation, the large amount of unstructured data and also to some extent a lack of trust in private companies to provide this kind of service. However, there is a trend in Europe showing an emerging political support from several countries to invest in health data hub and electronic health records [21]. Sharing of health data through the implementation of electronic health records across Europe will enable optimised use of health data to improve healthcare and outcomes for patients as well as promoting research. Furthermore, rare disease patients have expressed their willingness to share their own data for the benefit of others. Indeed, rare disease patients acknowledge the fact that the project in which they are participating will not necessarily have an impact on their quality of life but rather on that of future or younger patients affected. Helping patients affected by their disease in the future or patients affected by other diseases has proven to be the strongest incentive for patients to participate in research initiatives (CISRP 2017 [18, 37]). The fact that patients are motivated to enrol in clinical studies they believe to be scientifically or socially important has been demonstrated among wider patient populations [35].

However, rare disease patients’ willingness to share their data does come with specific conditions and requirements. For most patients, it is important to:

### Control how and for which purpose their health data will be shared

Being in favour of sharing data and calling for more control are not contradictory, they are clearly stated as two parallel requirements. Respondents clearly need to be at the centre of data-driven innovation and to be recognised as active agent in data sharing initiatives in which they participate. The current regulatory environment coupled with the trend in public debate sparkled by news on various data breaches, including on social media, are factors contributing to the need for greater control especially by respondents living in the European Union. According to the General Data protection Regulation (Article 6 (4); Recital 502,018), organisations that process personal data for research purposes may avoid restrictions on secondary processing and on processing sensitive categories of data including health data. However, patients’ request for control over their data build the case to enable patients to express preferences regarding the use of their data.

Results also show that views regarding the sensitivity of data, preferences in terms of frequency and means to be informed and trust in stakeholders vary significantly by sociodemographic profile. Echoing these trends, dynamic systems have started to emerge as tools that would enable to provide regular and accessible information to patients regarding the purpose and outcome of the projects whilst allowing patients to select and tailor their preferences related to when, how and who can use their data, thus respecting individual preferences with the possibility to amend these over time [41]. More specifically, the concept of dynamic consent has been recently tested and reviewed [5] offering the additional potential for improving research outcomes and providing the adapted and flexible system that will be much needed in view of the future technological and regulatory/legal changes in the European health systems. Person-centred approaches and digital solutions are also pushed forward in the Communication on the transformation of Health and Care in the Digital Single Market to organising health and care to allow citizens to actively engage in their health and access scientific information more easily [15].

### Minimise risks and respect concerns whilst promoting research

The perceived specific sensitive nature of genetic and genomic data and the additional vigilance to the way this data should be handled compared to other health data has been reported in previous qualitative studies ([31], RD-Connect Delphi exercise). This view is more nuanced among the present sample of respondents as information on disability is seen as the most sensitive among the list (51% compared to 49%). Furthermore, in their report “Genome sequencing: what do patients think” published in 2015, Genetic Alliance UK [23] states that 93% of the surveyed patients do welcome the sharing of their genomic data for research purposes. The Genetic Alliance UK report adds that patients consider a lack of genomic data sharing as an hindrance to scientific research progress which in turn would be counter-intuitive to their hope for a better quality of lives.

The perceived sensitivity of information on disability can be linked to discrimination rare disease patients are experiencing with regard to their condition on a daily basis, in particular in a school or work environment, which was widely reported in previous qualitative activities [31] and other Rare Barometer surveys [17]. Ethical and responsible data sharing should be enabled through widespread implementation of the IRDiRC recognised resource, the international charter of principles for sharing bio-specimens and data which provides guidance for effective legally- and ethically-grounded data sharing. Furthermore, several ongoing initiatives are testing the use of the blockchain technology to protect personal data [45]. Biotechnology companies are also using blockchain to share and protect genomic data (e.g. Genomes.io [24]).

### Increase transparency and improve communication

Ensuring patient trust and confidence in the different projects involving data sharing will help sustainable patient participation and increase the chance of successful outcomes for the project. In healthcare settings, it is also associated with better health outcomes in improving treatment adherence, for instance. The results clearly demonstrate that rare disease patients show various levels of trust in different actors and stakeholders who could be involved in data sharing platforms and initiatives. Trust in public bodies who are most of the time initiating and supervising data-sharing initiatives hardly reaches half of the respondents. This has to be considered in a context in which trust toward governments in general and among the general public [36] is low and decreasing. On average in OECD countries, in 2017, only 42% of citizens report having confidence in their government compared 47% in 2007. Lessons should be learned from the collapse of NHS England’s care data programme which was paused in 2014 and later abandoned largely due to a loss of public trust [41]. Also, the high level of trust towards healthcare professionals involved in rare disease patient’s daily care is important: data sharing initiatives would certainly benefit from involving general practitioners and other healthcare professionals in the management and communication of data sharing initiatives. Interestingly, the Caldicott report states that specific measures need to be taken to gain public trust including better technology standards, proper marketing of the benefits, an easy opt-out procedure, and a “dynamic consent” process [30]. Similarly, respondents expressed different levels of confidence on the ability of ethics committees to grant access to users of their data. Therefore, governance of data sharing initiatives and platforms should include a variety of actors to instigate confidence in the initiatives and ensure patient participation.

The good practices developed and implemented within the framework of the FP7-funded project RD-Connect for the governance of the platform provide confidence to patients and also researchers who deposit data in the platform. The project partners have developed a Code of Conduct to which users of the RD-Connect platform must adhere to in order to gain access. An additional safeguard is ensured by the data access committee who review all requests for access for the platform and to rule on circumstances where a user’s access may be revoked for lack of adherence to the Code of Conduct or other breach of best practice. This committee includes bioinformaticians, clinicians, researchers and patient representatives.

The survey has emphasised the need for rare disease patients to gain access to information related to their disease. It is important to enable rare disease patients to better understand their own health with easier access to information. The rarer the disease, the greater the need for patients - already experts on their disease - to continue to build knowledge on every aspect of their disease and enable them to share updated information with their peers. Scientists, clinicians, patients, industry and policy makers concerned with progress in rare disease research, healthcare and policy, ultimately share a similar goal, which is to ensure faster access to accurate diagnosis and improve healthcare. Therefore, impactful communication within the community needs to be made of strong and accessible common messages in order to break the siloed pattern inherent to rare disease data and expertise.

## Conclusion

The findings of this project add to the emerging literature about patient engagement in rare disease research and the value of, and barriers to, sharing data. The work of EURORDIS on this present survey on patients’ perspective and suggested recommendations can inform the moving landscape of data sharing and contribute to this paradigm shift of new norms and expectations.

Taking into account, i) the results of this survey, ii) previous qualitative studies and rare disease patients discussion groups, iii) the evidence-based policy work of EURORDIS, seven recommendations are detailed below. The goal of these recommendations is to inform and support stakeholders involved in data sharing to shape all relevant initiatives.

### Recommendation 1

Policy makers should ensure implementation of appropriate legislations at European and national levels and pursue efforts to foster cultural, technological and infrastructural changes to further develop international data sharing initiatives in health and research for rare diseases.

### Recommendations 2 and 3

Governing structures of data-sharing initiatives should:

- Develop and implement robust standards to ensure secure, ethical and responsible data sharing whilst putting in place safeguards around data protection;
- Include representatives from trusted advocacy organisations, i.e. patient organisations and non-profit organisations as well as clinicians and healthcare professionals.

### Recommendation 4

All stakeholders involved in data sharing initiatives need to promote the development of, and implement, dynamic systems enabling: i) the possibility to express different attitudes and preferences and ii) access to updated information on research outcomes to increase patient participation in research and stimulate data sharing whilst respecting patients’ preferences.

### Recommendation 5

All stakeholders involved in data sharing initiatives including healthcare systems and other relevant authorities should allocate resources at national and regional levels to enable the development of, and facilitate access to, relevant educational resources to enable informed choices for patients to share or not to share their health-related data.

### Recommendation 6

Funders and sponsors of data sharing activities should ensure that adequate financial resources are allocated to improve communication and increase transparency on the purpose and outcomes of data sharing initiatives to maximise the benefits of the project outcomes.

### Recommendation 7

Funders, clinicians and researchers need to emphasise potential health benefits of research studies and healthcare initiatives on future generations and other disease areas, as an incentive for wider participation in data sharing initiatives.

### Limitations

Although this is a significant, large scale survey there are several limitations that future researchers might need to take into account. Participants were identified through a large database of patients who have previously identified themselves as willing to take part in research and surveys to support the work of EURODIS, which might suggest a particular perspective about the value of research and patient participation. Patients with rare disease who are not on the Rare Barometer Voices database are a much harder to reach population. It is also important to note the over representation of female respondents in this sample, which highlights that men with rare disease or male family members are an important, yet under researched population. Although we recognise that these results might not be generalizable to all patients and all rare disease groups, this study represents an important step in understanding the views of those with rare disease and has led to recommendations to support future research and patient engagement.

## Additional files

Additional file 1: Profile of the respondents. (DOCX 16 kb)

Additional file 2: Relationship with rare diseases. (DOCX 15 kb)

Additional file 3: Repartition by group of diseases [19]. (DOCX 16 kb)

Additional file 4: Diseases with more than 20 respondents. (DOCX 15 kb)

Additional file 5: Repartition by country. (DOCX 17 kb)

## Data Availability

More detailed data, in particular socio-demographic breakdowns of the results, is available upon request.
