# Supplementary material for: Share and protect our health data: an evidence based approach to rare disease patients’ perspectives on data sharing and data protection - quantitative survey and recommendations
Source: Orphanet J Rare Dis. 2019 Jul 12;14:175. doi: 10.1186/s13023-019-1123-4 (PMC6625078; doi:10.1186/s13023-019-1123-4)
Supplement: Supplementary file 1 — Profile of the respondents. (DOCX 16 kb) [file 13023_2019_1123_MOESM1_ESM.docx]

**Additional file 1: Profile of the respondents**

|  | Number of people | % of responses |
| --- | --- | --- |
| Age (n = 2013) |  |  |
| 15-17 | 2 | 0% |
| 18-24 | 70 | 3% |
| 25-34 | 293 | 15% |
| 35-49 | 852 | 42% |
| 50-64 | 644 | 32% |
| 65 or older | 152 | 8% |
| Gender (n = 2013) |  |  |
| Female | 1534 | 76% |
| Male | 473 | 23% |
| Other | 6 | 0% |
| Country of residence (n = 2013) |  |  |
| European Union | 1775 | 88% |
| Outside the European Union | 238 | 12% |
| Diagnostic status (n = 2013) |  |  |
| Diagnosed | 1909 | 95% |
| Undiagnosed | 104 | 5% |
| Number of rare diseases (n = 1909) |  |  |
| 1 | 1664 | 87% |
| 2 | 174 | 9% |
| 3 | 44 | 2% |
| 4 | 13 | 1% |
| 5 or more | 14 | 1% |

In terms of repartition of the population, proportions are quite similar to the general population. A large part of the sample is living in the European Union. Female proportion is high (76/24) compared to the general population (52/48). This figure reflects the fact that the primary carer role for people living with rare diseases is primarily assumed by the mother (EURORDIS, 2017, 71% of parents living with a rare disease identify the mother as the main carer in the household). More than 90% of the sample is diagnosed and 5% are not diagnosed. These later can be either ‘Not yet diagnosed’ meaning that their disease has not been diagnosed because they have not been referred to the appropriate clinician due to misleading symptoms, or an unusual clinical presentation of a known rare condition or ‘Undiagnosed’ meaning that they are affected by a disease for which a diagnostic test is not yet available (EURORDIS, 2016).
