# Supplementary material for: Share and protect our health data: an evidence based approach to rare disease patients’ perspectives on data sharing and data protection - quantitative survey and recommendations
Source: Orphanet J Rare Dis. 2019 Jul 12;14:175. doi: 10.1186/s13023-019-1123-4 (PMC6625078; doi:10.1186/s13023-019-1123-4)
Supplement: Supplementary file 2 — Relationship with rare diseases. (DOCX 15 kb) [file 13023_2019_1123_MOESM2_ESM.docx]

**Additional file 2: Relationship with rare diseases**

| Are you a … (several answers possible) | | |
| --- | --- | --- |
| (n = 2321) | Number of people | % of responses |
| Parent of a child or adult with a rare disease | 642 | 32% |
| Sibling of a child or adult with a rare disease | 50 | 3% |
| Other family member | 86 | 4% |
| Patient | 1360 | 68% |
| Patient representative | 167 | 8% |
| Other | 16 | 1% |
| *Several answers possible, so percentage does not total 100%* | | |

The majority of the sample (68%) is composed of patients living with a rare disease and 32% of parents with a child or adult living with a rare disease. Among the total sample, 8% of the respondents are patient representatives, meaning that they are involved in advocacy activities related to rare diseases.
