# Supplementary material for: Share and protect our health data: an evidence based approach to rare disease patients’ perspectives on data sharing and data protection - quantitative survey and recommendations
Source: Orphanet J Rare Dis. 2019 Jul 12;14:175. doi: 10.1186/s13023-019-1123-4 (PMC6625078; doi:10.1186/s13023-019-1123-4)
Supplement: Supplementary file 3 — Repartition by group of diseases [19]. (DOCX 16 kb) [file 13023_2019_1123_MOESM3_ESM.docx]

**Additional file 3: Repartition by group of diseases**

In order to collect information on the disease, the Orphanet (the online reference portal for information on rare diseases and orphan drugs) inventory of diseases was used. If participants were diagnosed, they were able to select their condition from 14 318 modalities (or diseases). Diseases are grouped in of the 24 grouping of diseases for the European Reference Networks (see EURORDIS Proposal for grouping of diseases for ERNs in Evangelista et al., 2016). Among respondents, 1909 people were diagnosed and 1820 specified their disease. 1780 were then classified. The repartition into groupings is as follow:

|  | Number of people | % of responses |
| --- | --- | --- |
| Group of diseases represented in the sample  Multiple answer (n = 1780) |  |  |
| Rare Neurological Diseases | 222 | 12% |
| Rare Malformations, Developmental Anomalies & Rare Intellectual Disabilities | 195 | 11% |
| Rare and Undiagnosed Skin Disorders | 181 | 10% |
| Rare Connective Tissue & Musculoskeletal | 156 | 9% |
| Rare Immunodeficiency, Autoinflammatory and Autoimmune Diseases | 162 | 9% |
| Rare Pulmonary Diseases | 142 | 8% |
| Rare Neuromuscular Diseases | 149 | 8% |
| Rare Hereditary Metabolic Disorders | 126 | 7% |
| Rare Bone Diseases | 106 | 6% |
| Rare Endocrine Diseases | 92 | 5% |
| Rare Haematological Diseases | 62 | 3% |
| Rare Hepatic Diseases | 55 | 3% |
| Genetic Tumour Risk Syndromes | 45 | 2% |
| Rare Multisystemic Vascular Diseases | 39 | 2% |
| Rare and Complex Epilepsies | 35 | 2% |
| Adult Solid Rare Cancer | 38 | 2% |
| Rare Eye Diseases | 33 | 2% |
| Rare Renal Diseases | 18 | 1% |
| Rare Gastrointestinal Diseases | 24 | 1% |
| Rare & Complex Urogenital Diseases and Conditions | 13 | 1% |
| Rare Craniofacial and ENT Disorders | 9 | 1% |
| Rare Cardiac | 8 | 0% |
| Paediatric cancer | 1 | 0% |
| Total | 1911 | 107% |
