# Supplementary material for: Share and protect our health data: an evidence based approach to rare disease patients’ perspectives on data sharing and data protection - quantitative survey and recommendations
Source: Orphanet J Rare Dis. 2019 Jul 12;14:175. doi: 10.1186/s13023-019-1123-4 (PMC6625078; doi:10.1186/s13023-019-1123-4)
Supplement: Supplementary file 4 — Diseases with more than 20 respondents. (DOCX 15 kb) [file 13023_2019_1123_MOESM4_ESM.docx]

**Additional file 4: Diseases with more than 20 respondents**

| (n =1820) | Number of people | % of responses |
| --- | --- | --- |
| Other diseases | 1461 | 80% |
| Ehlers-Danlos syndrome | 73 | 4% |
| Sarcoidosis | 52 | 3% |
| Osteogenesis imperfecta | 42 | 2% |
| Meunière disease | 36 | 2% |
| Lupus erythematosus | 31 | 2% |
| Systemic sclerosis | 31 | 2% |
| Myasthenia gravis | 25 | 1% |
| Williams syndrome | 25 | 1% |
| Neurofibromatosis type 1 | 24 | 1% |
| Cystic Fibrosis | 20 | 1% |
| Because of rounding, percentage might not add up to exactly 100 | | |
