# Supplementary material for: Share and protect our health data: an evidence based approach to rare disease patients’ perspectives on data sharing and data protection - quantitative survey and recommendations
Source: Orphanet J Rare Dis. 2019 Jul 12;14:175. doi: 10.1186/s13023-019-1123-4 (PMC6625078; doi:10.1186/s13023-019-1123-4)
Supplement: Supplementary file 5 — Repartition by country. (DOCX 17 kb) [file 13023_2019_1123_MOESM5_ESM.docx]

**Additional file 5: Repartition by country**

| Which country do you live in? | | |
| --- | --- | --- |
| (n = 1997) | Number of people | % of responses |
| France | 240 | 12% |
| Spain | 238 | 12% |
| Germany | 195 | 10% |
| Italy | 170 | 8% |
| United Kingdom | 145 | 7% |
| Netherlands | 96 | 5% |
| Finland | 82 | 4% |
| Croatia | 71 | 4% |
| Belgium | 70 | 3% |
| Romania | 66 | 3% |
| Denmark | 60 | 3% |
| Portugal | 58 | 3% |
| United States of America | 44 | 2% |
| Czech Republic | 41 | 2% |
| Switzerland | 38 | 2% |
| Sweden | 32 | 2% |
| Hungary | 30 | 1% |
| Russia | 30 | 1% |
| Greece | 29 | 1% |
| Poland | 27 | 1% |
| Austria | 26 | 1% |
| Norway | 20 | 1% |
| Luxembourg | 19 | 1% |
| Ireland | 15 | 1% |
| Ukraine | 14 | 1% |
| Cyprus | 13 | 1% |
| Malta | 13 | 1% |
| Australia | 12 | 1% |
| Bulgaria | 11 | 1% |
| Mexico | 11 | 1% |
| Slovakia | 11 | 1% |
| Canada | 8 | 0% |
| Lithuania | 8 | 0% |
| Serbia | 8 | 0% |
| South Africa | 7 | 0% |
| Slovenia | 5 | 0% |
| Chile | 4 | 0% |
| India | 4 | 0% |
| Latvia | 4 | 0% |
| Malaysia | 4 | 0% |
| Bosnia and Herzegovenia | 3 | 0% |
| Argentina | 2 | 0% |
| Belarus | 2 | 0% |
| Brazil | 2 | 0% |
| Ecuador | 2 | 0% |
| Macedonia | 2 | 0% |
| Saudi Arabia | 2 | 0% |
| Algeria | 1 | 0% |
| Andorra | 1 | 0% |
| Central African Republic | 1 | 0% |
| Because of rounding, percentage might not add up to exactly 100%. | | |
